# An Intelligent Fire Warning Application Using IoT and an Adaptive Neuro-Fuzzy Inference System

**DOI:** 10.3390/s19143150

**Published:** 2019-07-17

**Authors:** Barera Sarwar, Imran Sarwar Bajwa, Noreen Jamil, Shabana Ramzan, Nadeem Sarwar

**Affiliations:** 1 Department of Computer Science and IT, The Islamia University Bahawalpur, Bahawalpur 63100, Pakistan; 2 Department of Computer Science, FAST – National University, Islamabad 44000, Pakistan; 3 Department of Computer Science, Govt. Sadiq College Women University, Bahawalpur, Punjab 54890, Pakistan; 4 Department of Computer Science, Bahria University, Lahore Campus, Lahore 54600, Pakistan

**Keywords:** multi-sensor, fire detection and warning system, adaptive neuro-fuzzy interference system (ANFIS)

## Abstract

In the recent past, a few fire warning and alarm systems have been presented based on a combination of a smoke sensor and an alarm device to design a life-safety system. However, such fire alarm systems are sometimes error-prone and can react to non-actual indicators of fire presence classified as false warnings. There is a need for high-quality and intelligent fire alarm systems that use multiple sensor values (such as a signal from a flame detector, humidity, heat, and smoke sensors, etc.) to detect true incidents of fire. An Adaptive neuro-fuzzy Inference System (ANFIS) is used in this paper to calculate the maximum likelihood of the true presence of fire and generate fire alert. The novel idea proposed in this paper is to use ANFIS for the identification of a true fire incident by using change rate of smoke, the change rate of temperature, and humidity in the presence of fire. The model consists of sensors to collect vital data from sensor nodes where Fuzzy logic converts the raw data in a linguistic variable which is trained in ANFIS to get the probability of fire occurrence. The proposed idea also generates alerts with a message sent directly to the user’s smartphone. Our system uses small size, cost-effective sensors and ensures that this solution is reproducible. MATLAB-based simulation is used for the experiments and the results show a satisfactory output.

## 1. Introduction

Fire and smoke kill more people every year than many other forces. While controlled fire serves us in so many instances, uncontrolled fire can be of harm, however, the rapid detection of fire and its control can save lives and property damage worth millions. Conventional and addressable are two main types of fire alarm systems, but unfortunately, these fire alarm systems often generate false alarms. The ratio of false alarm is higher in conventional alarm systems compared to addressable, but addressable alarm fire systems are more expensive. The most likely cause of a false warning is different for distinct types of detection systems, such as a smoke sensor often being activated falsely due to an environmental effect. So, there is a need for a cost-effective multi-sensors expert alarm system that is artificially trained and assists FDWS (fire detection and warning system) to make the right decisions and to reduce the number of false alarms. False alarm warnings are so common that London fire brigade alone is called out almost every 10 min to attend a false alarm causing them a loss of about £37 million per year. To achieve the aforementioned goal, in this paper, we introduced a home-based FDMS that uses a microcontroller Arduino UNO R3 (Arduino, Somerville, TX, USA) based on the atmega328p. It is easily available and programmed using the Arduino Software (IDE) with a set of cost-effective sensors. The proposed solution effectively uses a smoke sensor with flame sensors with a particular increase in room temperature; to further investigate the true presence of fire and to avoid false alarm, the FDWS is trained with a neuro-fuzzy designer. The purpose of this intelligent fire alarm system is to sense true occurrences of fire, alert the proper authorities, and notify the occupants via GSM to take necessary action immediately.

A false alarm can burden the fire brigade and can turn out to be a costly event; so many studies conducted to reduce them. Previous studies proposed different methods such as autonomous firefighting robots, fire alarm systems with notification appliances, and wireless warning systems. Fire alarm systems with notification appliances can be costly because they use visible and audible stimuli to notify residents. The primary objective of this paper is to develop a reproducible and economical solution with minimum false alarms and a system that alerts via GSM (global system for mobile communication). The innovative idea is to use neuro-fuzzy logic to design a smart alarm system. Our proposed system is ANFIS-simulated in MATLAB environment; the obtained results show effectiveness and the robustness with good performances compared with the FIS method (in Section 3). The ANFIS idea was originally proposed by Jang [1] in 1993. Typically, an ANFIS is a combination of a neural network and a fuzzy inference system (FIS) and is effective in making decisions.

### Related Works

This section discusses different AI techniques and other fire detection methods used in the past to mitigate risks of fire by early detection and reduce false warnings, but our main focus is ANFIS technology. Efforts were made for early fire detection and risk mitigation. Diverse technologies developed by researchers have been used such as fuzzy logic, neural networks, video-based techniques, Image Processing color-based fire detection methods, etc. Early Fire detection always has been an important research Topic for researchers. The idea of using multiple sensors was proposed by Faisal et al. [2]. The proposed wireless sensor network (WSN) consists of different sensors that share a single wireless network and used GSM. The proposed system results were tested in a smart home to reduce false warnings. Elias et al. also provided a solution using wireless sensor network that was embedded in a micro-controller board for fire hazard detection and fire monitoring purpose [3].

Hamdy et al. Built a “Smart Forest Fire Early Detection Sensory System (SFFEDSS)”, by combining the wireless sensor networks and artificial neural networks for the detection of forest fire [4].

Yu et al. [5] collected the sensor readings for smoke intensity, humidity, temperature to use it in fire detection using Feed-forward neural network approach. The disadvantage of a Feed-forward approach is it demands high processing at the node level resulting in a large amount of power consumption which reduces the lifespan of the node. Also, cluster head destruction in the fire badly affects the robustness of the system.

A system presented by Vikshant et al. works for detection of forest fire by combining wireless sensor networks (WSNS) [6,7,8,9] with fuzzy logic. Multi-sensors technology is used for detecting fire chances and early fire detection. Information gathered from different sensors such as heat, humidity and CO density light, will be sent on the cluster head using event detection mechanisms. Multiple sensors used to detect fire probability and direction are embedded in each node to reduce the false alarm rate and improve the efficiency [10]. A simple way to detect fire developed by Muralidharan et al. using Multiple sensors with the implementation of fuzzy logic and presented the obtained results in MATLAB [11].

In 2017, Yu-Liang Hsu. [12] developed a multi-sensor data fusion technology with artificial intelligence, wearable intelligent technology, and sensor fusion technology that can control home appliances and locate the position of home residents. It works in indoor environments. Similarly, a system was developed by Mirjana et al. which used an IoT concept for determining true fire presence according to the situation [10].

Robert et al. introduced a system using Arduino microcontroller and fuzzy logic technology in search of fire detection in automobile and to reduce its damage due to fire. Different sensors like temperature sensors, smoke sensors, and flame sensors were used. This system was tested on an average-sized car with 2 kg cylinder mounted behind the passenger’s rear seats [13].

J Olivares-Mercado [14] proposed a method of early fire detection by analyzing visual smoke characteristics such as color, dynamic texture, gray tones, etc. The system was tested using standard videos containing smoke.

JH Park. [15] proposed an early fire detection system for smart cities with a multifunctional artificial intelligence framework. The artificial intelligent framework includes an adaptive fuzzy algorithm, machine learning algorithms and Direct-MQTT based on SDN.

In this paper, ANFIS technology is used to design a fire detection control system and reduce false alarms. ANFIS technology has been used in mobile robot navigation [16], healthcare monitoring systems [17], air conditioning control [18], flood susceptibility modeling [19], and many other applications. In recent times, fiberoptic sensors were used for structural fire engineering [20], however, there is a need for true fire identification. IoT is successfully being used to achieve accuracy and efficiency in modern smart systems and has provided positive results as well [21,22,23,24]. This success of IoT was the motivation to design the proposed a smart and intelligent system for fire monitoring.

In the rest of the paper, Section 2 discusses the used approach and architecture of the proposed ANFIS-based decision support system for early fire identification and details of implementation. Section 4 discusses experimental settings with their results and this work is concluded in Section 5.

## 2. Materials and Methods

### 2.1. Adaptive Neuro-Fuzzy Inference System (ANFIS) Architecture

For generating an intelligent fire detection system that can monitor the parameters required for the actual presence of fire so that a false alarm can be decreased up to a minimum level, a combination of two important technologies fuzzy logic and artificial neural network (Ann) called the adaptive neural fuzzy interface system (ANFIS) can logically generate fuzzy rules according to training data to make the system robust. A fire detection system is developed using this aforementioned technology and presented in this paper to find the probability of fire. The ANFIS neural network works until the output matches the desired value for the given input. So, considering these abilities, an adaptive neuro-fuzzy interference system is used for detection of fire.

**Figure 1:**
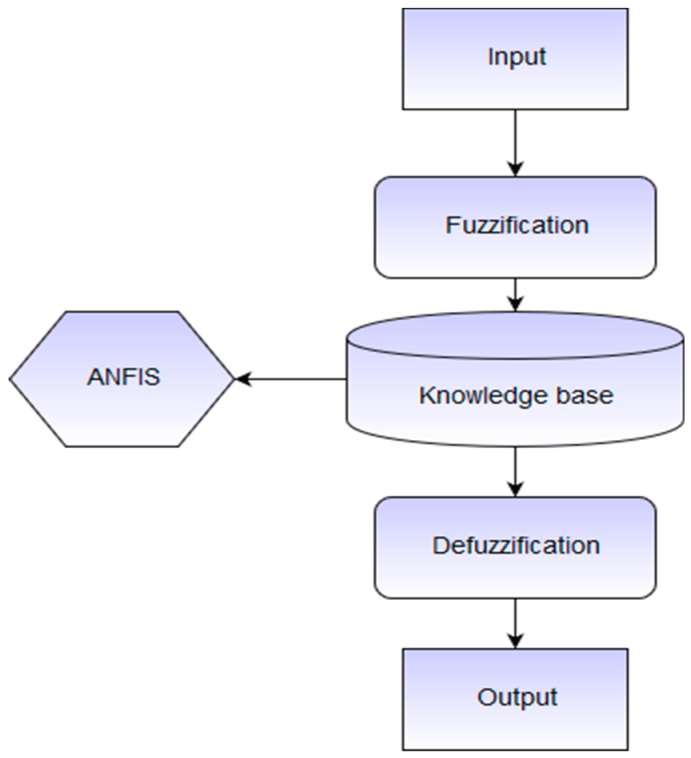
Typical Adaptive neuro-fuzzy Inference System (ANFIS) units.

ANFIS is a five-layer architecture that was developed in the early 1990s. The first layer is called the input layer. The second layer of ANFIS, called *inputmf,* is a fixed input membership function layer. The third layer depicts norms. The fourth layer, *outputmf,* is a fixed output membership function layer and the last is the output. The basic block diagram of ANFIS with input and output is illustrated in Figure 1.

Figure 1 contains different units of the ANFIS system. Input collected from sensors is trained in various steps. In the first step of fuzzification, raw data is collected, and the fuzzy interface system creates different rules artificially. The created rules are then further trained using the Sugeno method in MATLAB with the help of the artificial neuro-network. In the last step of de-fuzzification, the fuzzified data are again converted into raw variables. The main goal of this entire process is to minimize human effort and overcome manual errors.

There is a need to prioritize the actual parameters of fire so that false alarms could be reduced. To solve this particular problem, a fire monitoring system should be developed to monitor these parameters in real-time and quick action should be taken to reduce fire damage and save human life. The proposed Sugeno-based adaptive neuro-fuzzy interference system decides the presence of fire according to fuzzy rules and vital parameters collected from different sensors.

The designed real-time system collects data like temperature, smoke, humidity, and flame presence to provide the updated status to the owner using the GSM module.

### 2.2. Architecture of Proposed FDWS

**Figure 2:**
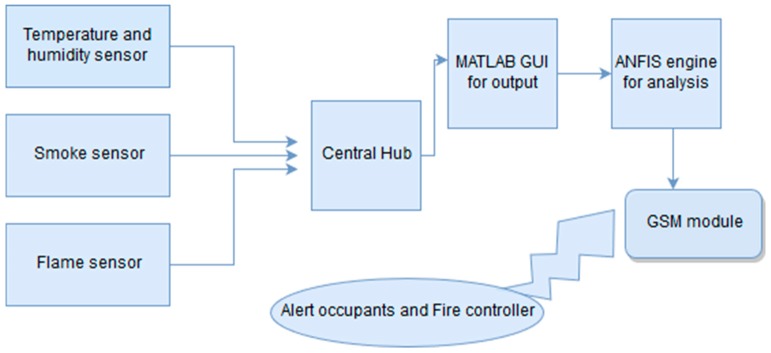
The architecture of the fire alarm system.

The architecture of the proposed fire detection and warning system is illustrated in Figure 2. The combination of software and hardware together create an automatic fire detection system. The system has different sensors such as a smoke sensor, temperature and humidity sensor, and a flame sensor. These sensors collect data from sensor nodes and then transmit it to the GUI in MATLAB. The data gathered from sensors is then provided as a raw data to fuzzy logic as a linguistic variable which is trained in adaptive neuro-fuzzy system to detect fire status. If the parameters show that the probability of fire is critical, then a message will be sent through GSM regarding the fire condition to the fire controllers and the house owner.

The whole system consists of two phases. The first phase of the hardware design includes the development of sensor nodes and the other phase consists of a MATLAB simulation. Both phases are further described in detail.

#### 2.1.1. Hardware Development in Proposed FDWS 

In this phase, we designed sensor nodes for fire monitoring employing multi-sensors such as temperature, humidity, smoke, and flame. An Arduino UNO atmega328p micro-controller is used to embed the sensors.

**Figure 3:**
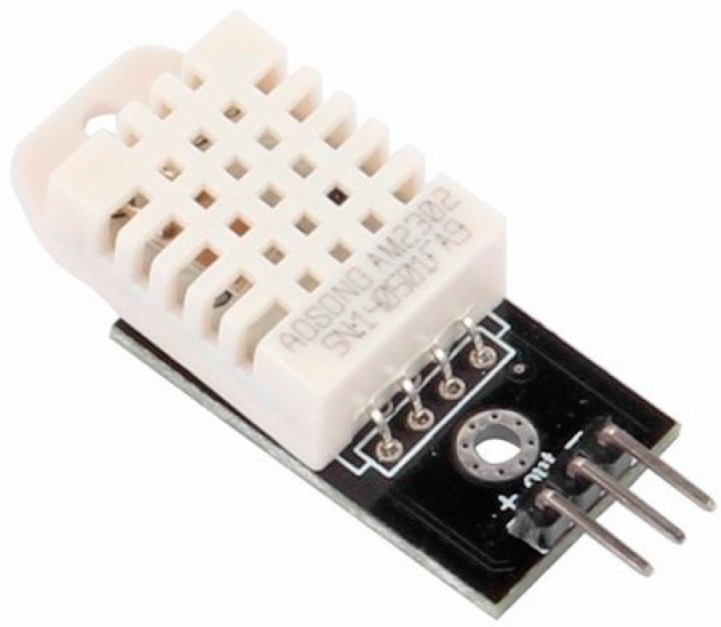
Temperature and humidity sensor used in the proposed system.

For gathering heat and humidity measurements, DHT22 is used which gives us two important measurements required for a smart fire monitoring system. It gives an output in degrees Celsius for temperature and percentage of humidity. This sensor is shown in Figure 3.

**Figure 4:**
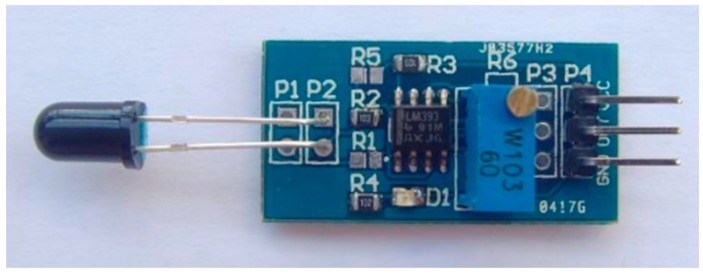
Flame sensor used in the proposed system.

The used flame sensor detects the flame at the range of 3 feet and at a 60 degree angle. The LED light shows the presence of fire. Figure 4 represents the flame sensor used in the proposed system.

**Figure 5:**
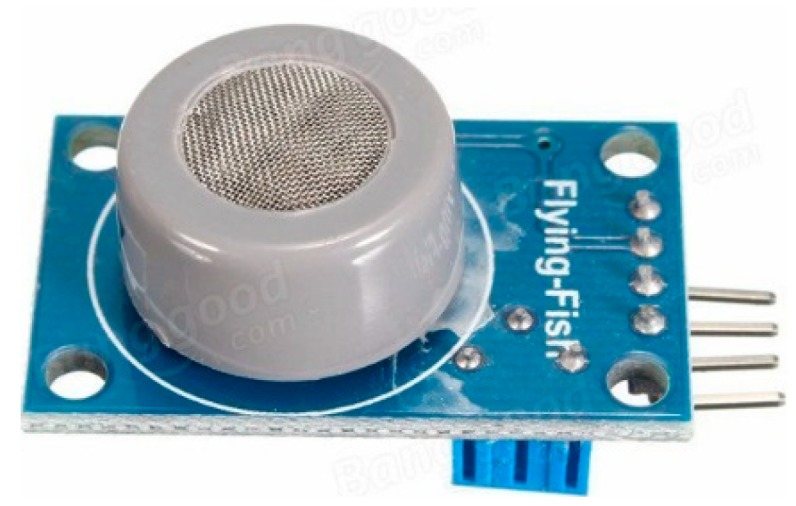
Smoke sensor used in the proposed system.

The MQ-7 Gas Sensor (see Figure 5) is used for the proposed system which is sensitive to carbon monoxide. Carbon monoxide results in a burning process. Its output boots with the concentration of CO level. It Can detect CO anywhere from 20 to 2000 ppm.

All these sensors are attached to the Arduino UNO ATmega328p board.

#### 2.1.2. PLX-DAQ

After successful configuration with Arduino UNO microcontroller, the PLX-DAQ Macro is used to acquire sensor data from the Arduino UNO to a Microsoft Excel sheet. We just downloaded the PLX-DUQ version 2 and established a connection with Arduino with the simple connection setting like baud rate set at 9600, and port 3 is selected in PLX-DAQ window.

#### 2.1.3. Coding in Arduino IDE

Arduino version 1.8.9 is used to program the hardware configuration of sensors. Coding is done in C language and built in libraries like *dht.h* for temperature and humidity sensor and *MQ7.h* for the gas sensor is used. The downloaded code is customized a little bit to get the desired results. The Arduino UNO is a simple way of communication between computer and microcontroller. The AT MEGA328p connects the UNO serial port, e.g., COM3 with the computer USB port which appears as a virtual COM in the PLX-DAQ. It is simple to use and just need to define connection settings to connect it with an Arduino. The embedded program code in the Arduino UNO board acquires sensor readings and represents it in the PLX-DAQ spreadsheet, as presented in Table 1.

The sample real-time data gathered for experimentation using Arduino and PLX-DAQ is also shown in Table 1 in an Excel spreadsheet.

#### 2.1.4. Gathering Sensor’s Data for Input Datasets

The training dataset is required for ANFIS training in MATLAB to train the proposed FDWS reading of the sensor data gathered in real-time situations under different scenarios like reading in normal conditions, severe conditions, and critical severe conditions. The actual data gathered from smoke, temperature, humidity, and flame sensors to train ANFIS data is acquired in an Excel sheet under all scenarios. The collected data helps in training the ANFIS. Table 2 shows the collected data.

In the above table, the real-time data are gathered using different sensors to find the expected input datasets for ANFIS for the proposed FDWS. The data is gathered in two different experiments. The first column of time interval represents the period of time of the fire to occur, and the second column represents the change rate of temperature in °C. The third column represents the humidity change rate and the fourth is for the smoke change rate in ppm in the presence of fire. In the first iteration of the experiment, when there is no fire present, the change rate of the temperature, humidity and smoke are not changing and are hence, zero. During the second iteration with the presence of fire, the rate of temperature is increased to 2 °C, the humidity is changed to 2% and the 3.8 ppm smoke rate is changed. In the same way, the results of the second experiment are collected and shown in Table 3.

The results of experiments used to calculate the universe of discourse for CR-temp, CR-humidity, and CR-smoke are illustrated in Table 4.

#### 2.1.5. MATLAB Simulation in Proposed FDWS 

**Figure 6:**
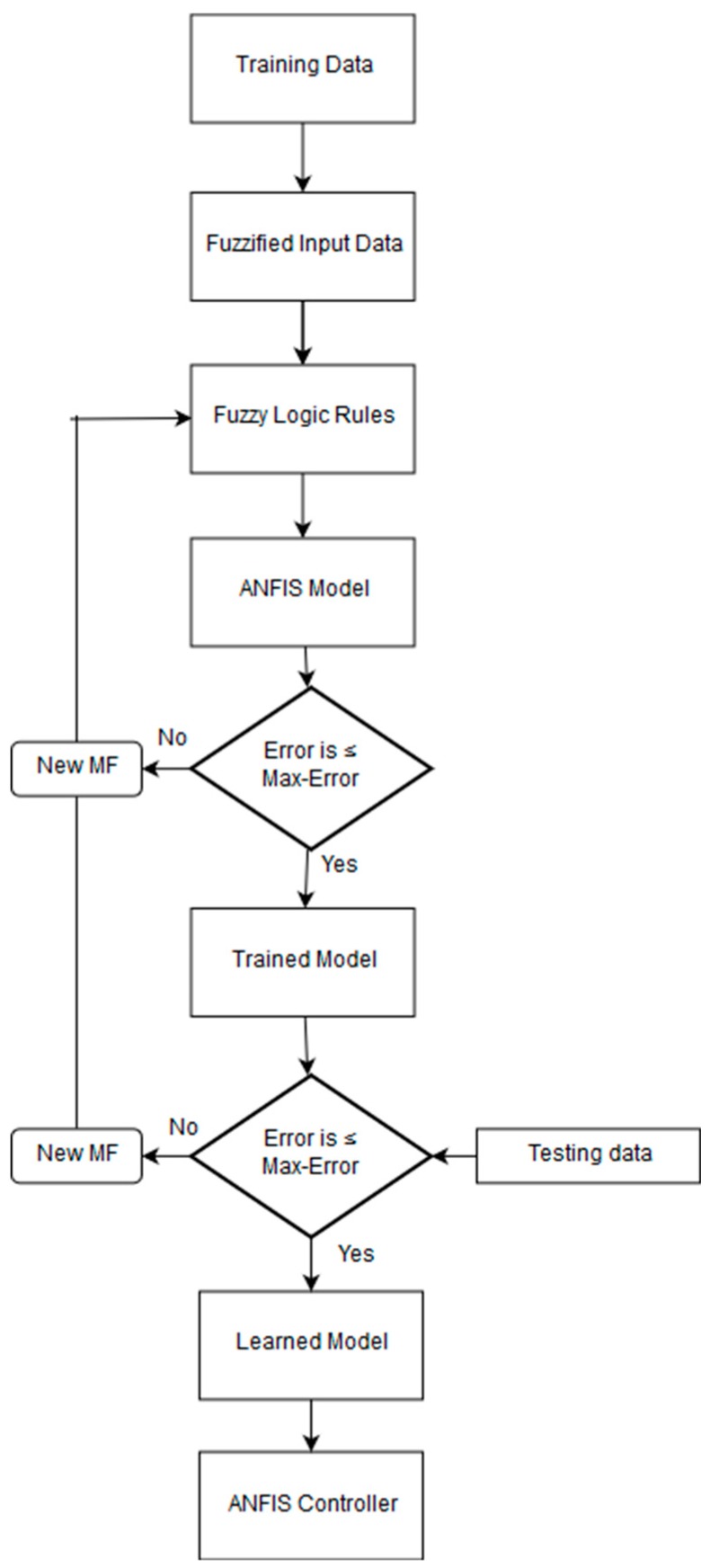
Training process.

The simulation of the proposed system is done in MATLAB. An Excel file has been created that shows various vital parameters of fire presence with reference to time. It receives data from sensor nodes and continuously displays it in real-time using PLX-DUQ. The graph of each input shows variations of data inputs at that time. A set of scenarios like no fire, medium fire, and critical fire were chosen. *ANFISedit* command is used to open the ANFIS training window or ANFIS is also found in the fuzzy control toolbox. We stored that data in a fuzzy file and used it for the training of the ANFIS with 100 epochs. Another set of data was provided to further test it and reduce the error rate. All steps of the training process are depicted in Figure 6.

Once the membership functions are designed, the ANFIS-generated rules are applied.

**FDWS rules for Flame Sensor:** The ANFIS-generated rules are applied only when the flame is detected, otherwise, FDWS keeps sensing the surroundings until the fire is detected.

The flame Sensor takes Boolean values (Yes, No/0,1) to detect the presence of fire. In Table 5, the first column ensures the presence of a flame, the second column is “repeat first step” repeated until a fire is detected. Column three representing “go to next step” means if a flame is present, it will jump to the next step in further evaluating the severity of fire by checking other important parameters. The presence of flame is necessary to go to the next step.

## 3. ANFIS Implementation for Proposed FDWS 

Jang (1993) was the father of ANFIS which was based on the Sugeno fuzzy logic model. Generally, ANFIS combines the least square estimation and back-propagation for membership function measurements. The integration of fuzzy logic with neural networks increases the learning ability of neural network and fuzzy system. Each generated rule is a linear combination of inputs and a constant term. The final output is calculated by weighing the output of each rule. Rules should not be more than output member functions. The proposed system has four inputs, but for simplicity purposes, two inputs *x* and *y* and one output *z* are used. The rules based on the Sugeno if-then rules are as follows:

Rule 1: If x is a1 and y is b1, then f1 = p1A + q1B + r_1_ (1)

Rule 2: If x is a2 and y is b2, then f2 = p2A + q2B + r_2_ (2)

**Figure 7:**
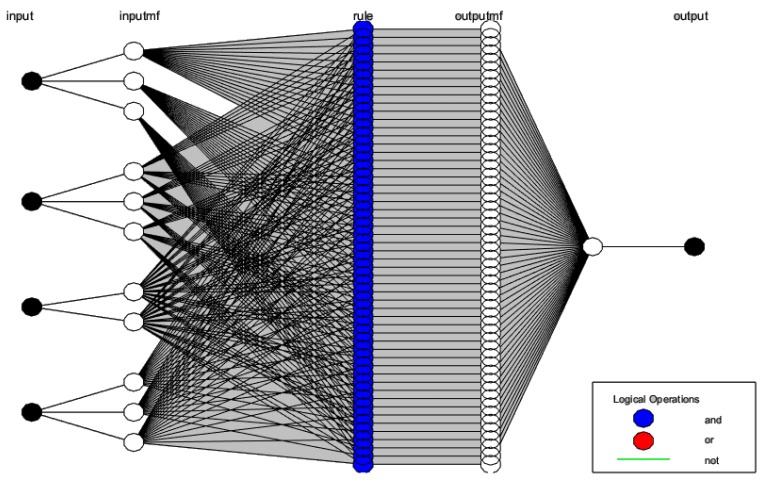
The ANFIS structure.

The ANFIS structure model is illustrated in Figure 7. It has five layers. The input layer called layer 0 is not part of the five layers. The presented system has four inputs (CR-temperature, CR-humidity, Time (the rate at which input changes), and CR-smoke).

Layer 1 is the fuzzification layer. Each node of layer 1 produces the membership grades of a linguistic label as the generalized bell function.

(3) $$bell\left(x;a,b,c\right)\;=\;\frac{1}{1+{\left|\left(x-c\right)/a\right|}^{2b}}$$

where {*a*, *b*, *c*} is the premise parameter set. The shape of the bell function varies as the parameter values change.

(4) $$O^{2}\;=\;w_{i}=\mu a_{i}\left(x\right)\mu b_{i}\left(y\right),\mathrm{where}\;i\;=\;1,2.$$

The product *μa_i_*(*x*).*μb_i_*(*y*) represents the strength of a rule. *x* and *y* in Equation (4) represent inputs with value *a_i_* and *b_i_*, respectively.

Layer 3 normalizes the firing strength of all the rules by calculating the ratio using Equation (5) provided that the summation of the strength of all the rules with 1 data is 1.

(5) $$O^{3}={\bar{w}}_{i}=\frac{w_{i}}{\sum w_{i}},\mathrm{where}\;i\;=\;1,2.$$

Layer 4 nodes are called adaptive nodes. These nodes compute the parameter function.

(6) $$O^{4}={\bar{w}}_{i}f_{i}=\left({\bar{w}}_{i}(p_{i}*x_{1}+q_{i}*x_{2}+b_{i}\right))$$

{*p_i_* *q_i_* and *b_i_*} are consequent parameters set. The parameters in this layer refer as consequent parameters.

Layer 5 is the output layer and summation of all the outputs coming from layer 4.

(7) $$O^{5}=\sum {\bar{w}}_{i}f=\frac{\Sigma w_{i}f_{i}}{\sum w_{i}},\;\mathrm{where}\;i\;=\;1,2$$

**Figure 8:**
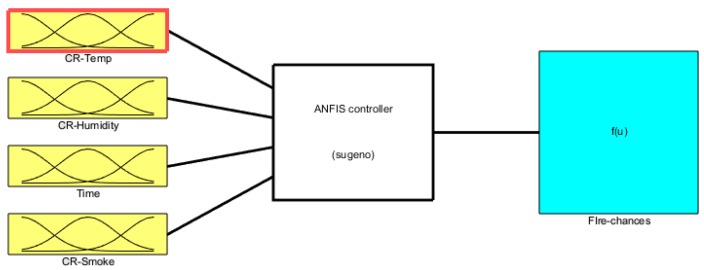
ANFIS Sugeno engine.

The raw data given as input is then converted into the desired output using the ANFIS technique. ANFIS weighted that input data and mapped it to output for fire detection; in real-time, the smart fire detection and monitoring system is combined with fuzzy interference and neural network. The four vital inputs are checked using the Gaussian form of membership functions. The developed fuzzy interference model is shown in Figure 8.

**Figure 9:**
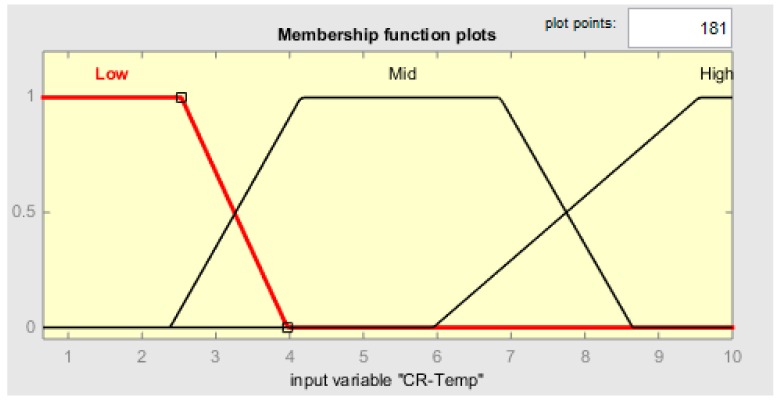
CR-Temp MF plot.

**Figure 10:**
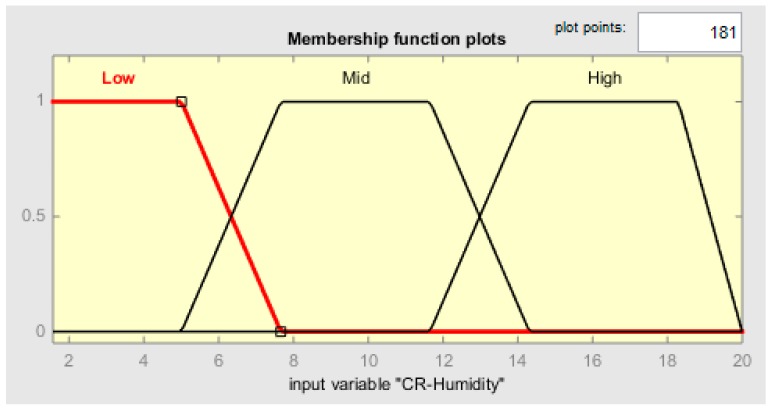
MF plot for CR-Humidity.

**Figure 11:**
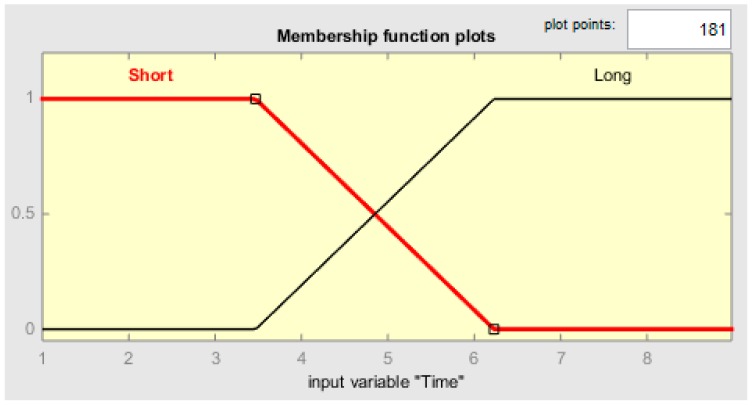
MF plot for TIME.

**Figure 12:**
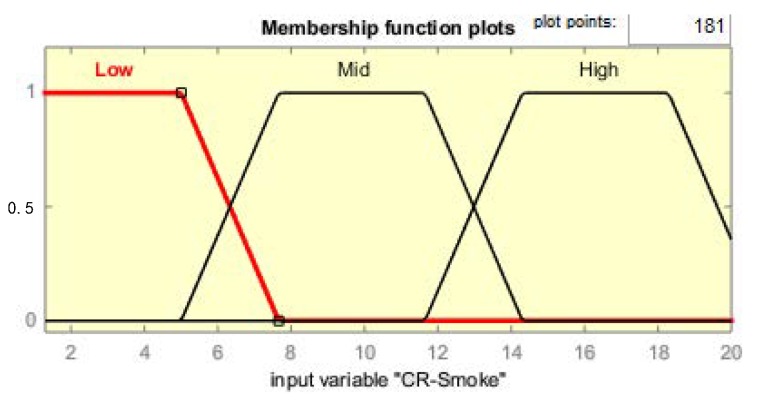
MF plot for Smoke.

Each membership function plot is given in Figure 9, Figure 10, Figure 11 and Figure 12, respectively, for CR-temperature (change rate of temperature), CR-humidity, CR-smoke, and Time variations.

### ANFIS Generated Rules in MATLAB Rules Editor

**Figure 13:**
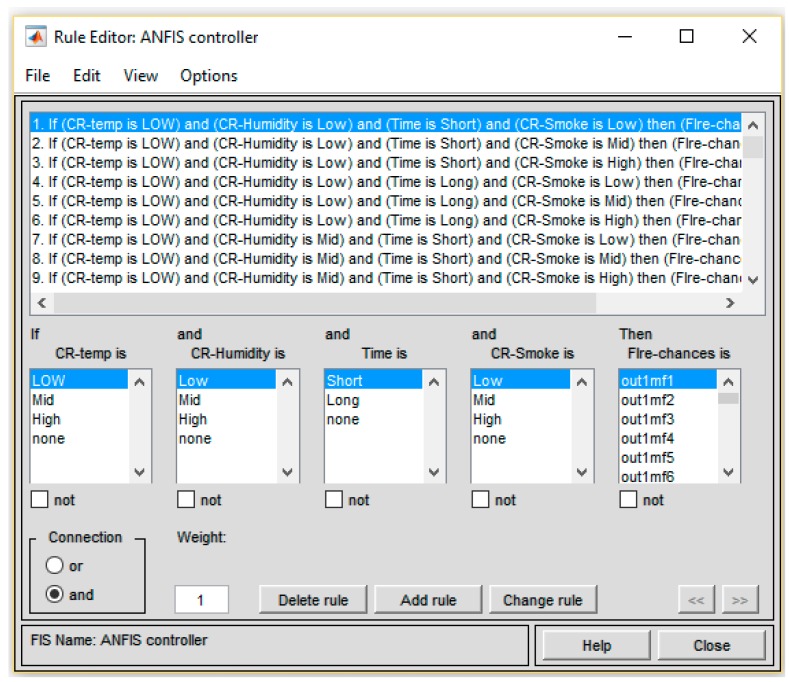
ANFIS generated rules.

After defining Input membership functions and output, the training process automatically generates the Rules. The training process requires the implementation in the Sugeno method to train the fuzzy linguistic labels. The *Training.dat* file, which contains training data collected from sensors, is provided to ANFIS. After successful training, it generated 54 rules. The generated rules are shown in Figure 13. It shows different parameters of fire based on the *AND* operator. For ANFIS training command to work, the initial FIS structure must have each rule with a different output membership function; that is, the number of output membership functions must equal the number of rules.

## 4. Results and Discussion 

### 4.1. Proteus Simulation for Proposed FDWS

**Figure 14:**
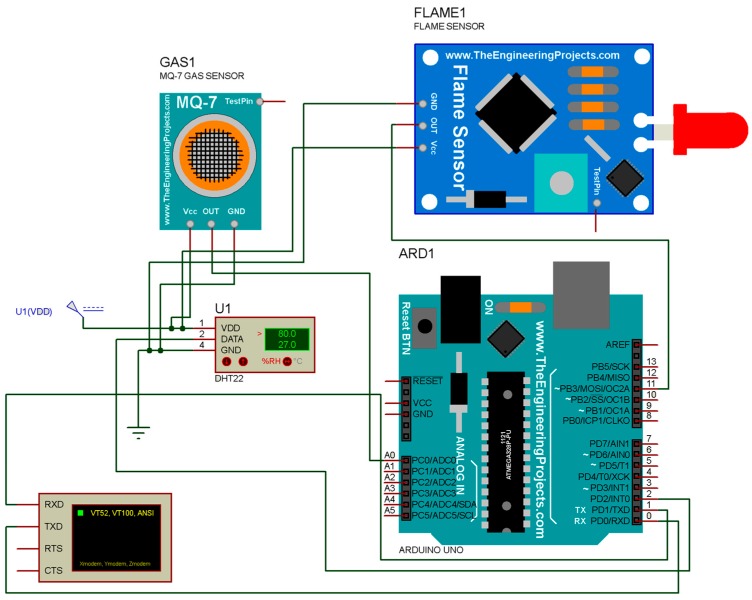
Proteus simulation.

The simulation of hardware used in the proposed system is done in Proteus 8.1 using DH22 as a temperature and humidity sensor; for gas concentration, an MQ7 CO detector sensor is used. A flame sensor is also shown in Figure 14. A schematic diagram drawn in Proteus is shown in Figure 14 and is used for simulation for real-time values to further test the performance of a hardware module.

Experimental analysis of different membership functions based on surface results is given below in Table 6, Table 7, and Table 8.

### 4.2. MATLAB ANFIS Simulation

**Figure 15:**
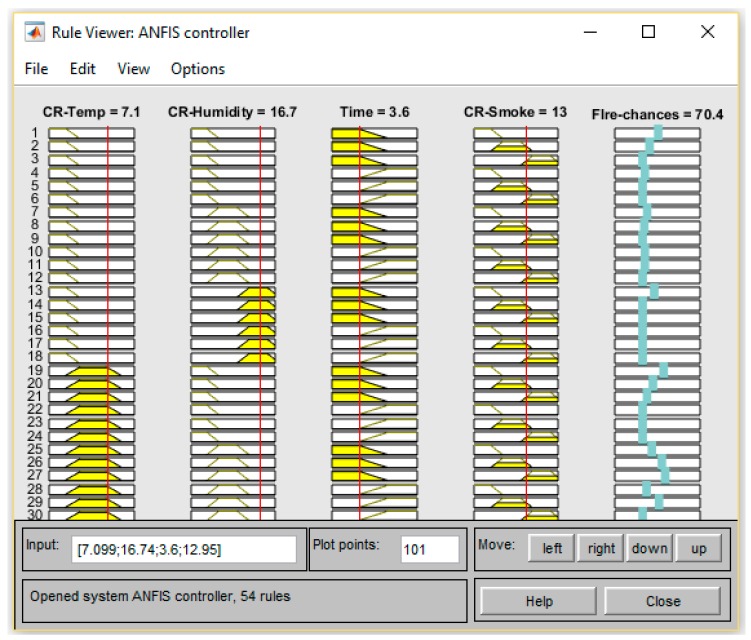
ANFIS rules viewer.

**Figure 16:**
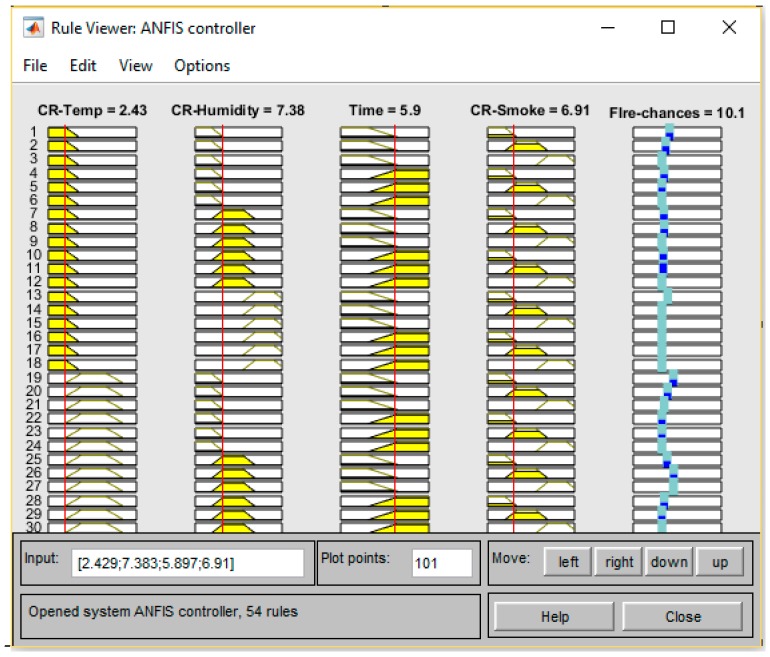
ANFIS output for different inputs.

**Figure 17:**
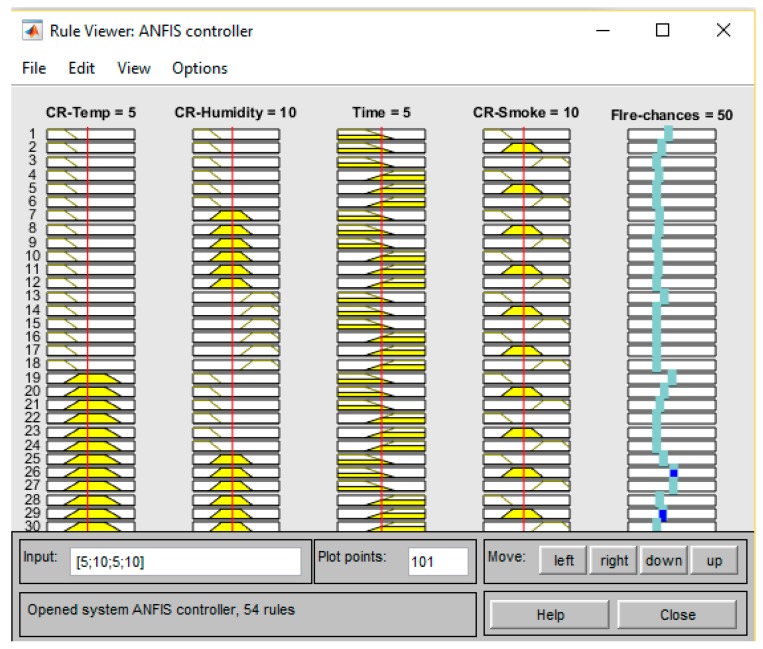
Fire-Chances with input variations.

The training and testing datasets help the FDWS to be more robust and self-adaptive to minimize false warnings. The probabilities of fire presence for different input values after training are shown in Figure 15, Figure 16 and Figure 17, respectively. The presented results in Figure 15 show a high probability of fire, so an urgent message is delivered to occupants via GSM.

**Figure 18:**
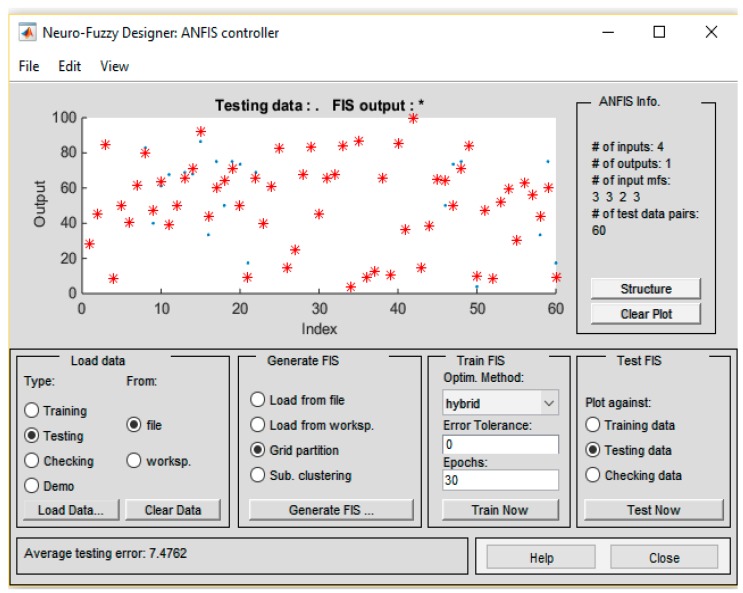
Average testing results.

The testing data which is shown in Figure 18 demonstrates that the average testing error 7.4762 RMSE (root mean square error) was observed. This shows it is a good model to predict fire chances.

**Figure 19:**
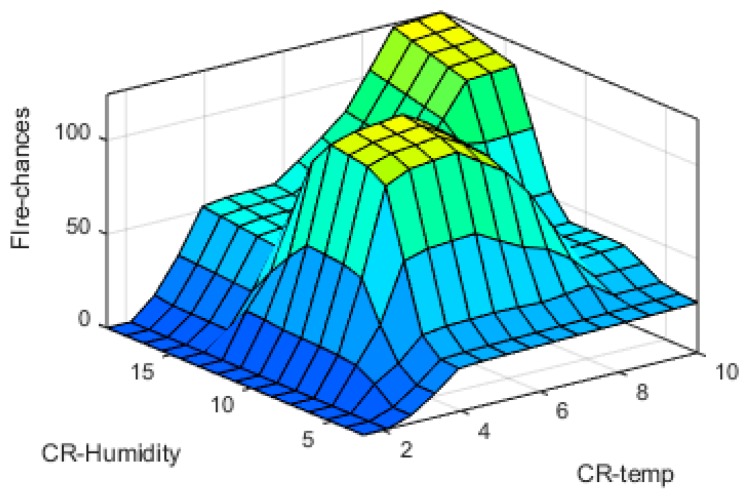
CR-Humidity v/s CR-Temp surface plot.

**Figure 20:**
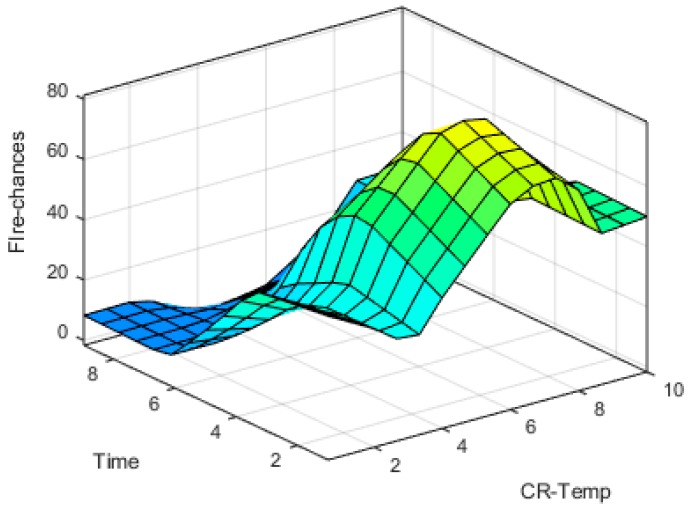
Time vs. CR-Temp surface plot.

**Figure 21:**
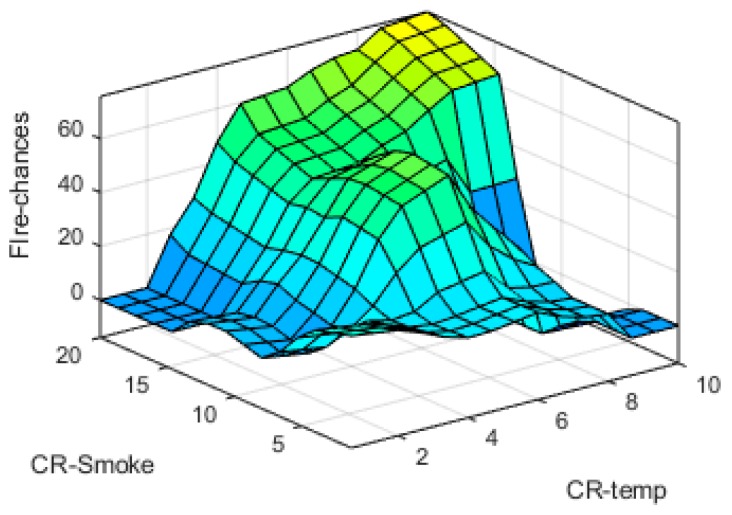
CR-Smoke vs. CR-Temp surface plot.

**Figure 22:**
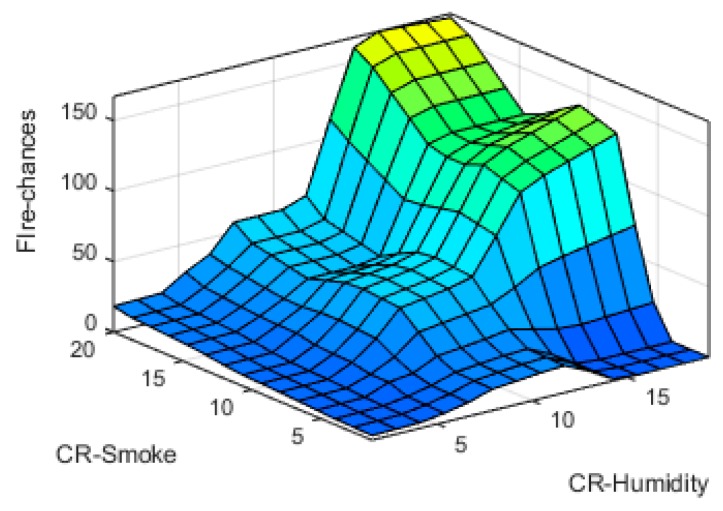
CR-smoke vs. CR-Humidity surface Plot.

The input-output fuzzy membership functions are analyzed in detail. The generated 3D graphs show the variation of fire chances with reference to the changes in different parameters at input sides such as variation with temperature and humidity, time and humidity, smoke and temperature, and smoke and humidity are shown in Figure 19, Figure 20, Figure 21 and Figure 22, respectively.

### 4.3. Contribution to Knowledge

The comparison between the method used in the proposed work and the other popular method FIS (fuzzy interface system) is described in Table 9. ANFIS has all the characteristics of FIS and uses a neural network to train the FIS; itallows the membership functions from FIS to be adapted.

To predict fire chances, the results from the FIS alone are compared with the ANFIS results. After that, these results are compared with the actual case from the experiments in order to validate the model and determine the superiority of ANFIS over FIS techniques. In Table 10, the comparison between FIS and ANFIS is illustrated, in the 10th iteration, FIS showed Mid fire chances while ANFIS showed Low as it was in the actual case. From Table 10, we can infer that ANFIS is more effective and strongly recommended for prediction of fire chances.

## 5. Conclusions and Future Work

This paper proposed an intelligent and smart fire warning system for smart buildings. This system not only analyses the fire presence, but also notifies the concerned people for severe fire chances in case of an emergency or critical situation. ANFIS architecture model makes the proposed system more efficient, robust and reliable; and reduces false alarms; the proposed system used easily available, lightweight and cost-effective sensors and is more reliable than conventional fire detection systems. This system can be used at the commercial level and results are reproducible. Further advancement in the proposed system can be achieved by researching more into precise and lightweight sensors that provide more accurate signals for analysis. Furthermore, the use of IoT (internet-of-things) can enhance the system by talking with various other devices and smart systems like sending the message to smart gas meters to stop the supply of gas in critical conditions, etc. This system is particularly designed for indoors, as the flame sensor is sensitive to sunlight and, secondly, the reading and training data may differ in open areas, but the minor change in training can overcome this problem.

## Figures and Tables

**Table 1:** A sample of experimentally measured data in real-time.

Time	Flame Presence	Smoke	Humidity	Temperature
5:19:17 PM	No Flame	3.04 ppm	22.00%	35.00 °C
5:19:18 PM	No Flame	3.04 ppm	22.00%	35.00 °C
5:19:19 PM	No Flame	3.04 ppm	22.00%	35.00 °C
5:19:21 PM	No Flame	3.04 ppm	23.00%	35.00 °C
5:19:22 PM	No Flame	3.04 ppm	22.00%	35.00 °C
5:19:24 PM	No Flame	3.04 ppm	23.00%	35.00 °C
5:19:25 PM	No Flame	3.04 ppm	23.00%	35.00 °C
5:19:27 PM	No Flame	3.04 ppm	22.00%	35.00 °C
5:19:28 PM	No Flame	3.04 ppm	22.00%	35.00 °C
5:19:30 PM	No Flame	3.04 ppm	22.00%	35.00 °C
5:19:31 PM	No Flame	3.04 ppm	22.00%	35.00 °C
5:19:33 PM	No Flame	3.04 ppm	22.00%	35.00 °C
5:19:34 PM	No Flame	3.04 ppm	22.00%	35.00 °C
5:19:36 PM	No Flame	3.08 ppm	23.00%	36.00 °C
5:19:37 PM	Flame detected!	3.12 ppm	24.00%	36.00 °C
5:19:39 PM	Flame detected!	3.31 ppm	24.00%	36.00 °C
5:19:40 PM	Flame detected!	3.38 ppm	25.00%	36.00 °C
5:19:42 PM	Flame detected!	3.34 ppm	25.00%	36.00 °C
5:19:43 PM	Flame detected!	3.31 ppm	24.00%	36.00 °C

**Table 2:** C-R-Temp, C-R-Temp, and time of fire detection with experiment 1.

	Experiment 1			
Sr.no	Time Interval (Minute)	C-R Temp (°C)	C-R Humidity (%)	C-R Smoke (ppm)
1	2.8	0	0	0
2	3	2	−2.8	3.8
3	4.1	3.8	−1.3	4
4	4	6.4	10.8	10.2
5	2	3	7.1	4.12
6	3.3	4.6	9.5	8.56
7	1.4	2	5	4
8	2.44	5	7.6	10

**Table 3:** C-R-Temp, C-R-Temp, and time of fire detection with experiment 2.

Experiment 2			
Time Interval (Minutes)	CR-Temp (°C)	CR-Humidity (%)	CR-Smoke (ppm)
1.5	0.6	0.8	0.9
1.16	2.2	1.9	3.3
56 s	1.2	2.2	3
2.38	3	2.5	6
2.12	1	2	2
1.56	3	6	5.5
2	2.8	8	6
2	3.2	6	6.73

**Table 4:** The universe of discourse for each membership function.

Variable	CR-Temp (°C)	CR-Humidity (%)	CR-Smoke (ppm)	Fire-Chances	Time (Min)
Low	0–4	0–8	0–8	0–30	-
Mid	2.5–8.5	5–14	5–15	30–60	-
High	5.5–10	12–20	12–20	60–100	-
Short	-	-		-	0–5
Long	-	-		-	4–9

**Table 5:** Rules for FMWS (according to flame presence).

Flame	Repeat First Step	Go to Next Step
Flame Detected	No	Yes
Flame is not detected	Yes	No

**Table 6:** Experimental analysis of CR-Temp(C).

Sr. No	CR-Temp (C)	Fire Chances	Condition
1	0–2	0–20	Normal
2	2–5	20–40	Critical
3	5–10	>60	Severely critical

**Table 7:** Experimental analysis of CR-Humidity (%).

Sr. No	CR-Humidity	Fire Chances	Condition
1	0–8	0–20	Normal
2	4–14	20–40	Critical
3	>15	>60	Severely critical

**Table 8:** Experimental analysis of Smoke (ppm).

Sr. No	Smoke (ppm)	Fire Chances	Condition
1	0–8	0–20	Normal
2	4–14	20–40	Critical
3	12–20	>60	Severely critical

**Table 9:** Comparison of two related technologies FIS and ANFIS.

Properties	Methods	
	FIS	ANFIS
Non-linear characterization	Yes	Yes
Automatic training	No	Yes
Knowledge needed for modeling biological phenomenon	A lot of human effort Requires	Do automatically from Datasets
Automatic Adaptation of output and membership functions	No	Yes

**Table 10:** Comparison of original case, ANFIS case, and FIS case.

Sr. No.	CR-Temp (°C)	Flame Presence	CR-Humidity (%)	CR-Smoke (ppm)	Time (Min)	Chances of True Fire (%)	ANFIS Case	FIS Case	Original Case	Accuracy (%)
1	1.53	1	7.85	2.1	2.53	8.4	Low	Low	Low	100%
2	5.1	1	9.9	10.3	5	50	Mid	Mid	Mid	100%
3	3.66	1	9.6	9.5	2.53	40	Mid	Mid	Mid	100%
4	7.31	1	12.9	14.4	1.99	61.2	High	High	High	100%
5	7.89	1	14.3	13.4	7.3	82.9	High	High	High	100%
6	1.87	1	3.01	5	1.49	14.3	Low	Low	Low	100%
7	2.71	1	5.18	5.2	2.51	45	Mid	Mid	Mid	100%
8	8.1	1	7.35	14.2	1.39	67.7	High	High	High	100%
9	6.69	1	17.2	12.9	2.35	83.4	High	High	High	100%
10	1.27	1	14.8	2.4	2.35	45	Low	Mid	Low	100%
11	5.48	1	10	10.3	1.39	65.9	High	High	High	100%
12	8.9	1	16.1	15.2	4.04	83.9	High	Mid	High	100%
